# Acute kidney injury after arterial switch operation: incidence, risk factors, clinical impact – a retrospective single-center study

**DOI:** 10.1080/0886022X.2023.2167661

**Published:** 2023-01-24

**Authors:** Anton Puzanov, Vadym Tkachuk, Andriy Maksymenko

**Affiliations:** Ukrainian Children’s Cardiac Center, Kyiv, Ukraine

**Keywords:** Arterial switch operation, acute kidney injury, neonates, pediatric cardiac surgery

## Abstract

**Background:**

This retrospective study aimed to determine the incidence, risk factors, and outcomes of acute kidney injury (AKI) in neonates following the arterial switch operation (ASO) for transposition of great arteries (TGA).

**Methods:**

Retrospective review of medical data of children who underwent ASO in 2019–2020 in the Ukrainian Children’s Cardiac Center.

**Results:**

76 consecutive neonatal patients were included, 48 developed AKI after ASO (51.7%), and 24 – had severe AKI (25.8%). Severe AKI development was associated with longer cross-clamp time: 82 (61–127) versus 73.5 (53–136) in the non-severe AKI group (*p* = 0.02). 76 min of cross-clamp time were defined as a threshold value for increased severe AKI risk, OR 4.4 (95% CI: 1.5 – 13, *p* = 0.01). Higher lactate levels during cardiopulmonary bypass (CPB) increased severe AKI development risk, OR 1.5 (95% CI: 1.0 − 2.0, *p* = 0.03). Children with severe AKI had prolonged mechanical ventilation, longer time to negative fluid balance, and higher postoperative day 3 (POD3) Inotropic Score (IS). Only one patient required peritoneal dialysis.

**Conclusions:**

In our study, 51.7% of patients developed AKI after ASO, 25.8%–severe AKI. Prolonged cross-clamp time and higher lactate levels during cardiopulmonary bypass increased the risk for severe AKI development. The development of AKI was associated with prolonged mechanical ventilation, longer time to negative fluid balance, higher POD 3 Inotropic Score.

## Introduction

Acute kidney injury (AKI) is common in children after cardiac surgery. It occurs in 15–64% of patients after congenital heart surgery [1], as cardiopulmonary bypass (CPB) is one of the main causes of this complication [2]. AKI has a negative impact on patient treatment: it lengthens stay in the intensive care unit (ICU) [3–4] and increases mortality [5].

Patients with transposition of great arteries (TGA) are a special interest group in studying kidney function. They are newborns with cyanosis who require complex cardiac surgery with long CPB. It greatly increases the risk of developing AKI [6].

There are a few studies describing the incidence of AKI among patients after total repair of TGA – arterial switch operation (ASO). However, the characteristics and complexity of patients included in these studies are heterogeneous. According to this data, AKI develops in 20–50.7% of cases [2–4].

The Ukrainian children’s cardiac center is a leading Ukrainian hospital in the treatment of congenital heart diseases. We perform surgical treatment of nearly 1000 infants younger than 3-years-old yearly; 200 of them are newborns. Children with TGA are admitted to the Center in the first minutes after delivery, and ASO is performed in the first hours of the neonate’s life. Nearly 50 neonates underwent ASO in the first days or hours of life yearly. This approach allows us to exclude preoperative factors’ impact (ICU stay, sepsis, mechanical ventilation, etc.) [7].

Our aim was to determine the incidence of AKI among neonates following ASO in our Center, identify potential risk factors for the development of this complication, and assess the impact of AKI on the postoperative course.

## Material and methods

A retrospective cohort study of consecutive patients who underwent ASO surgery in the 2019–2020 years in the Ukrainian children’s cardiac center was performed. Patients with TGA (both with intact ventricular septum and ventricular septal defect (VSD)) and Tausig-Bing anomaly were included. We collected and analyzed medical data during pre-, intra-, and postoperative treatment stages, from admission to ICU until the patient’s ICU discharge/death.

The study was approved by the Institutional Review Board (IRB) of the Ukrainian Children’s Cardiac Center (Extract from protocol №6 dated 22.06.2021, outgoing letter 1010/21-8-1).

### Preoperative data

Information, collected before ASO, included: age at the time of surgery, serum creatinine, total serum protein level, and usage of prostaglandins and inotropes. Anatomical variants, included in the analysis: TGA with and without VSD, Tausig-Bing anomaly, and patients with and without aortic arch pathology.

### Intraoperative data

We analyzed the duration of CPB and aortic cross-clamp time, amount of red blood cells (RBC) transfused, highest arterial blood lactate level during CPB, and fluid balance data.

### Postoperative data

Values of postoperative course were analyzed, including intensive care unit length of stay (ICU LOS), duration of ventilation, fluid balance in the first three postoperative days (POD) (day of surgery counted as day 0), need in peritoneal dialysis (PD) catheter placement. As a measure of inotropic support, we used the Inotrope score (IS), calculated by Wernovsky et al. [8]

Kidney function was evaluated using the pRIFLE scale [9]. This scale is shown to be the most sensitive test for detecting early AKI in pediatric cardiac patients, especially in the infant age group. It is also effective in the early identification of AKI in pediatric ICU patients [10]. Lowest serum creatinine before surgery was set as the baseline. Patients were classified as having AKI based on the pRIFLE criteria using the highest post-operative creatinine at the PICU. Four categories according to pRIFLE were used to group patients:

AKI 0 – creatinine level after surgery <150% compared to baseline.
AKI 1 – creatinine level after surgery 150–200% compared to baseline.
AKI 2 – creatinine level after surgery 200–300% compared to baseline.
AKI 3 – creatinine level after surgery >300 compared to baseline, or need in kidney replacement therapy (peritoneal dialysis).
AKI 2 and AKI 3 were considered severe AKI in the risk factors analysis.

### Data analysis

Statistical analysis for this data was conducted using Microsoft Excel, StatSoft, Inc. (2014) STATISTICA, version 12. Continuous variables were described as medians with ranges. Categorical variables were presented as frequencies and percentages. Baseline comparisons between patients from the non-severe AKI and the severe AKI groups were performed using the chi-square or Mann–Whitney *U*-test depending on the distribution. Univariate and multivariate logistic regression were performed to identify risk factors for developing severe AKI. To find a cut-point that maximizes the variable’s ability to differentiate the non-severe AKI from the severe AKI endpoint the ROC curve analysis was performed and the Youden index (J) was computed. A *p*-value of less than 0.05 (two-sided) was considered statistically significant.

## Results

**Figure 1.**
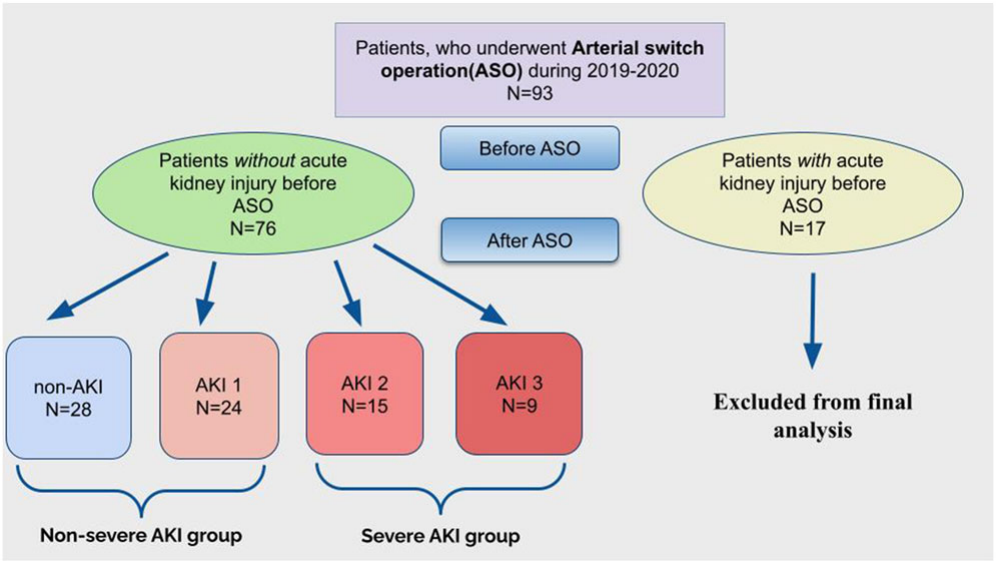
Groups of patients after ASO.

During 2019–2020 93 ASO procedures were performed for neonates and infants with TGA and Tausig-Bing anomaly. A total of 65 AKI cases were diagnosed. In 17 patients with AKI developed before surgery, they were excluded from the final analysis. In 48 (51.7%) patients AKI first developed after ASO. 24 patients (50%) were classified as AKI 1, 15 patients (31.3%) as AKI 2, and 9 patients (18.7%) as AKI 3. Severe AKI (stages 2 and 3) developed in 25.8% of patients. Two groups of patients were formed: the non-severe AKI group − 52 patients (28 with no AKI and 24 with AKI 1) and the severe AKI group − 24 children (Figure 1).

### Preoperative characteristics

Patients’ data before ASO is presented in Table 1. Presence of VSD, coarctation of the aorta, and Tausig-Bing anomaly were not associated with an increased risk of AKI.

**Table 1.** Patients’ data before ASO.

Variable	Non-severe AKI group *N* = 52	Severe AKI group *N* = 24	*p*-value
Age at time of surgery, days	1 (0–54)	1 (0–10)	0.94^a^
Lowest serum creatinine, μmol/L	64.7 (14.7 − 121.1)	59.2 (22.6 − 78.7)	0.03^a^
Lowest diuresis rate, mL/kg/hour	1.5 (0 − 3.7)	1.1 (0 − 4.2)	0.57^a^
Lowest serum protein level, g/L	47 (40.1 − 58.7)	48 (42.9 − 66.7)	0.43^a^
Highest arterial blood lactate level, mmol/L	2.7 (1.1–9)	2.5 (1.1 − 7.9)	0.48^a^
Lowest arterial blood pH	7.3 (7.07 − 7.45)	7.27 (7.15 − 7.5)	0.44^a^
Nadir SpO2, %	73 (16–91)	71 (12–88)	0.65^a^
Need for inotropic support, %	16 (30.7%)	11 (37.5%)	0.23^b^
Prostaglandin usage	29 (55.7%)	17 (70.8%)	0.21^b^
Presence of ventricular septal defect	9 (17.3%)	6 (25%)	0.44^b^
Presence of coarctation of the aorta	5 (9.6%)	2 (8.3%)	0.86^b^
Tausig-Bing anomaly	5 (9.6%)	2 (8.3%)	0.86^b^

Data are in median (range) or *n* (%).

^a^Mann-Whitney *U*-test.

^b^Pearson’s Chi-square test.

There were no significant preoperative differences between the two groups, except for creatinine values. Lowest preoperative creatinine was significantly lower in the severe AKI group: 59.2 (22.6 − 78.7) versus 64.7 (14.7 − 121.1) in the non-severe AKI group (*p* = 0.03).

#### Intraoperative data

Intraoperative data of patients from two groups are presented in Table 2.

Aortic cross-clamp time was significantly longer in the severe AKI group, 82 (61–127) versus 73.5 (53–136) in the non-severe AKI group (p = 0.02). Maximal lactate level during CPB was significantly higher in the severe AKI group: 4.7 (2–10) versus 3.3 (0.9–8.5) in the non-severe AKI group. Cardiopulmonary bypass duration, red blood cells transfusion volume, intraoperative Inotrope Score, and fluid balance were not different in the two groups.

**Table 2.** Intraoperative data.

Variable	Non-severe AKI group *N* = 52	Severe AKI group *N* = 24	*p*-value
Cardiopulmonary bypass time, min	146 (110 – 252)	164 (111–308)	0.13^a^
Cross-clamp time, min	73.5 (53–136)	82 (61–127)	0.02^a^
Red blood cells transfusion volume, mL	90 (0–190)	92.5 (20–190)	0.99^a^
Highest lactate during cardiopulmonary bypass, mmol/L	3.3 (0.9 − 8.5)	4.7 (2–10)	0.03^a^
Inotrope score	5 (0–12)	5 (3 − 7.5)	0.57^a^
Fluid balance, mL	50 (-100–225)	95 (-50–200)	0.20^a^

Data are in median (range).

^a^Mann-Whitney *U*-test.

#### Postoperative data

Information about the postoperative course of the patient is presented in Table 3.

**Table 3.** Postoperative data in two groups.

Variable	Non-severe AKI group *N* = 52	Severe AKI group *N* = 24	*p*-value
ICU length of stay, days	8 (3–71)	10 (5–61)	0.22^a^
Ventilation duration, hours	70 (12–1554)	129 (43–1090)	0.01^a^
Need for PD catheter placement	2/52 (3.8%)	3/24 (12.5%)	0.34^b^
RBC transfusion volume, mL	30 (0 – 500)	40 (0–240)	0.28^a^
Delayed sternal closure	0 (0%)	4 (16.7%)	0.01^b^
Mortality	0/52 (0%)	1/24 (4.2%)	0.14^b^
Fluid balance data			
Fluid balance POD 0 (mL)	−12 (-280–+150)	+54 (-170–+200)	0.02^a^
Fluid balance POD 1 (mL)	−75 (-260–+137)	−28 (-200–+230)	0.05^a^
Fluid balance POD 2 (mL)	−106 (-487–+115)	−125 (-440–+195)	0.67^a^
Fluid balance POD 3 (mL)	−73 (-352–+207)	−20 (-306–+218)	0.55^a^
Inotrope score (IS)			
IS POD 1	5 (0 − 7.5)	5 (3 − 7.5)	0.26^a^
IS POD 2	4 (0 − 7.5)	5 (3 − 7.5)	0.07^a^
IS POD 3	3 (0 − 7.5)	5 (0 − 7.5)	0.03^a^

ICU: intensive care unit; PD: peritoneal dialysis; RBC: red blood cells; POD: postoperative day; IS: Inotrope Score.

Data are in median (range) or *n* (%).

^a^Mann-Whitney *U*-test.

^b^Pearson’s Chi-square test.

Patients in the severe AKI had a longer duration of mechanical ventilation: 129 h (43–1090) versus 70d (12–1554) in the non-severe AKI group (*p* = 0.01). Only one patient in the severe AKI group required peritoneal dialysis. Significantly more patients in the severe AKI group had an open sternum after surgery: 4 (16.7%) in the AKI group versus 0 in the non-AKI group (*p* = 0.01). Fluid balance tended to be less restrictive in the first 2 days in the severe AKI group. In the next 2 days, this difference was not significant. Inotrope score (IS) was significantly higher at POD 3 in the severe AKI group: 5 (0 − 7.5) versus 3 (0 − 7.5) in the non-severe AKI group (*p* = 0.03).

#### Possible risk factors evaluation

We performed a univariate logistic regression analysis to identify potential risk factors for severe AKI development. Cross-clamp time, highest lactate value during CPB, IS POD 3 were analyzed, as there was a significant difference in their median values. Results are presented in Table 4.

**Table 4.** Results of univariate logistic regression analysis.

Variable	Odds ratio	95% CI	*p*-value
Risk factors for severe AKI			
Cross-clamp time, min	1.03	1.0 − 1.05	0.03
Highest lactate during CPB, mmol/L	1.5	1.0 − 2.1	0.01
IS POD 3, 1 unit increase	1.4	1.0 − 1.8	0.02
Cross-clamp time > 76min	4.4	1.5 − 13	0.01

Prolonged cross-clamp time was associated with an increased risk for severe AKI, OR 1.03 (95% CI: 1.0–1.05, *p* = 0.03). When Youden indexes were calculated, a threshold value of 76 min of cross-clamp time was found to discriminate the risk of severe AKI development (Sensitivity 75%, Specificity 59.6%), with OR 4.4 (95% CI: 1.5–13, *p* = 0.01). An association between the highest lactate level during CPB and increased risk for severe AKI development was found, OR 1.5 (95% CI: 1.0–2.1, *p* = 0.01). Increased IS POD3 was associated with severe AKI development, OR 1.4 (95% CI: 1.0–1.8, *p* = 0.02).

Multivariate logistic regression analysis was performed to identify independent risk factors of severe AKI development. Results are presented in Table 5.

**Table 5.** Results of multivariate logistic regression analysis.

Variable	Odds ratio	95% CI	*p-*value
Risk factors for severe AKI			
Cross-clamp time, min	1.03	1.0 − 1.06	0.05
Highest lactate during CPB, mmol/L	1.5	1.0 − 2.0	0.03

Multivariate logistic regression showed no association between an increased IS POD3 and severe AKI. Cross-clamp time was found to be a significant independent risk factor for severe AKI development, OR 1.03 (95% CI:1.0 − 1.06). Highest lactate during CPB was also found to be a significant independent risk factor for severe AKI development, OR 1.5 (95% CI:1.0 − 2.0).

## Discussion

To our knowledge information about the prevalence of AKI after ASO in developing countries is limited. We present our experience from a main Ukrainian pediatric cardiosurgical center. In our institution usual practice is to perform ASO in the first days and hours of life (median age of surgery 1 day in our cohort) [7,11]. This led to the fact that previously identified preoperative AKI risk factors like age, weight, and preoperative drugs [6,12] were not found to be significantly associated with the development of AKI in our study.

There is a big discrepancy between CPB and cross-clamp time impact in articles, dedicated to post-cardiac surgery AKI. The deleterious effect of CPB and cross-clamp on renal function is multifactorial and well-known and they are regarded as potentially modifiable risk factors [13,14]. CPB provokes AKI development, mostly due to kidneys' intraoperative hypoperfusion, non-pulsatile blood flow, and release of inflammatory mediators [15]. In adults both CPB and cross-clamp, times are independent AKI risk factors [16,17]. In the pediatric population, a threshold of 120 min of CPB was shown to increase the risk of AKI after cardiac surgery [5]. However, practically all ASO patients usually have at least 2 h of perfusion. Previous studies of AKI in patients after ASO did not find significant correlations between CPB and cross-clamp times and AKI development risk [1,3,4]. Basu suggested, that it shows limitations of applying data drawn from all-surgical and all-lesion types to specific surgical lesions [2]. However, in our study cross-clamp time was an independent predictor of severe AKI development, which is an original finding in our study. We proposed cutoff value of cross-clamp time for severe AKI risk based on ROC-curve analysis, although with low sensitivity and specificity values.

Duration of cross-clamp may be increased in case of complex heart surgery, including ASO with VSD closure or aortic arch repair. However, in previous studies, any association between these anatomical variants and increased AKI risk was not shown, as well as in our paper [1,3,4]. On the other hand, the relatively small number of patients enrolled could affect the results. Further research with larger groups is required, as well as multicenter studies.

Serum creatinine and creatinine clearance remain to be most precise and widespread AKI diagnostic tools in children. Zappitelli showed, that even a small rise in creatinine after cardiac surgery can predict AKI development [18]. He also found, that AKI most often develops in the first 4 days after surgery. However, the serum creatinine of newborns does not yet reflect neonatal but maternal serum creatinine levels [19]. It is reflected by the fact that creatinine level is significantly lower in the AKI group in our study. Although statistically significant, this finding reflects the methodology problem and not clinical relevance. This may have an impact on the accuracy of AKI diagnosis in neonates. Further research is required, to include maternal creatinine levels in the analysis and evaluate its impact on AKI diagnostic accuracy.

The positive fluid balance increases ICU LOS [3]. Usually, all patients after cardiac surgery are treated with diuretics (furosemide most often). The other widespread option is peritoneal dialysis (PD), being a safe and effective method of renal replacement therapy in cardiac neonatal ICU. In many centers, the insertion of a PD catheter at the end of the operation is common practice. It was shown that early initiation of PD allowed superior fluid management in neonates after cardiac surgery [20]. Early initiation of PD should be strongly considered in neonates at high risk of AKI and fluid overload after surgery [21]. In our cohort more PD catheters were placed in the severe AKI group patients, although not statistically significant. The main indication was usually ascites as a consequence of inadequate fluid management, and PD was initiated if the diuresis rate remained unsatisfactory. In our study only one patient in severe AKI group required dialysis. Different researchers evaluated the effectiveness of prophylactic PD catheter placement in cardiac surgery patients [22]. However, results are controversial, and the benefit of prophylactic catheter insertion depends on surgery severity and heart disease type. Generally, the final decision depends on the surgeon’s opinion and the institution's protocols.

In our study, severe AKI group patients needed relatively higher inotropic support (measured using an Inotropic score). Inotropic and vasoactive drugs are usually used in the early postoperative treatment of neonates after complex cardiac surgery, and associations between their doses and AKI have been studied. In Schoenmaker’s research paper patients with severe AKI had higher vasoactive-inotropic scores 24 and 48 h after surgery [4]. It was shown by Kumar, that higher IS was associated with a higher risk of AKI after pediatric heart surgery [23]. Davidson found that a higher VIS at 48 h after cardiothoracic surgery was most significantly associated with poorer outcomes – prolonged mechanical ventilation, and ICU LOS but had no association with peak creatinine or time to negative fluid balance [24]. Further studies are needed to define the use of quantitative indicators of inotropic support, like IS or VIS, in the prediction of AKI development in neonates after cardiac surgery.

## Study limitations

Our study was conducted only in one center, which limits its results. We have no data on a patient’s kidney function later in life. Further research is needed to evaluate the impact of neonatal surgery on the risk of chronic kidney disease later. In many cases, ASO was performed in the first hours of a patient’s life due to our Center's surgical policy [7]. In such cases, the only creatinine measurement obtained before surgery was set as a baseline. Maternal creatinine data was not available at the moment of the study, so it is unclear how it impacts the accuracy of AKI diagnosis in neonatal patients. Further studies could be conducted to include maternal creatinine levels in the analysis.

## Conclusion

In our retrospective single-center study, AKI incidence among neonates after ASO was 51.7%, and the severe AKI incidence (AKI 2 and 3)−25.8%. We have not found any predictors of AKI before surgery, which may be related to the relative homogeneity of the group (in terms of age) and minimal time between childbirth and ASO performed (only a few hours in some cases). Patients with severe AKI had longer cross-clamp times and higher lactate levels during CPB. Cross-clamp time and higher lactate levels during CPB were found as significant risk factors for severe AKI development. Development of AKI was associated with longer ventilation time and time to negative fluid balance, higher IS POD3. Only one patient in the severe AKI group required dialysis. Further studies are needed to search for possible predictors, early AKI diagnostic tools, and maternal creatinine impact on diagnosis accuracy.

## Data Availability

The datasets used and/or analyzed during the current study are available from the corresponding author on reasonable request.

## References

[1] Basu RK, Andrews A, Krawczeski C, et al. Acute kidney injury based on corrected serum creatinine Is associated with increased morbidity in children following the arterial switch operation. Pediatr Crit Care Med. 2013;14(5):e218–e224. 23439467 10.1097/PCC.0b013e3182772f61

[2] Basu RK, Devarajan P, Wong H, et al. An update and review of acute kidney injury in pediatrics. Pediatr Crit Care Med. 2011;12(3):339–347. 21057358 10.1097/PCC.0b013e3181fe2e0b

[3] Harmer MJ, Southgate G, Smith V, et al. Acute kidney injury and short-term renal support in the post-operative management of neonates following repair of transposition of the great arteries. Prog Pediatr Cardiol. 2019;52:26–32.

[4] Schoenmaker NJ, Weeda JA, van der Palen RL, et al. Acute kidney injury after the arterial switch operation: incidence, risk factors, and outcomes. Cardiol Young. 2022;32(5):794–799. 34350824 10.1017/S1047951121003176

[5] AlAbbas A, Campbell A, Skippen P, et al. Epidemiology of cardiac surgery-associated acute kidney injury in neonates: a retrospective study. Pediatr Nephrol. 2013;28(7):1127–1134. 23519522 10.1007/s00467-013-2454-3

[6] Park SK, Hur M, Kim E, et al. Risk factors for acute kidney injury after congenital cardiac surgery in infants and children: a retrospective observational study. PLOS One. 2016;11(11):e0166328. 27832187 10.1371/journal.pone.0166328 PMC5104485

[7] Chasovskyi K, Fedevych O, Vorobiova G, et al. Arterial switch operation in the first hours of life using autologous umbilical cord blood. Ann Thorac Surg. 2012;93(5):1571–1576. 22459547 10.1016/j.athoracsur.2012.01.104

[8] Wernovsky G, Wypij D, Jonas RA, et al. Postoperative course and hemodynamic profile after the arterial switch operation in neonates and infants. Circulation. 1995;92(8):2226–2235. 7554206 10.1161/01.cir.92.8.2226

[9] Akcan-Arikan A, Zappitelli M, Loftis L, et al. Modified RIFLE criteria in critically ill children with acute kidney injury. Kidney Int. 2007;71(10):1028–1035. 17396113 10.1038/sj.ki.5002231

[10] Lex DJ, Tóth R, Cserép Z, et al. A comparison of the systems for the identification of postoperative acute kidney injury in pediatric cardiac patients. Ann Thorac Surg. 2014;97(1):202–210. 24206964 10.1016/j.athoracsur.2013.09.014

[11] Fedevych O, Chasovskyi K, Vorobiova G, et al. Open cardiac surgery in the first hours of life using autologous umbilical cord blood. Eur J Cardiothorac Surg. 2011;40(4):985–989. 21353580 10.1016/j.ejcts.2011.01.011

[12] Chiravuri SD, Riegger LQ, Christensen R, et al. Factors associated with acute kidney injury or failure in children undergoing cardiopulmonary bypass: a case-controlled study. Paediatr Anaesth. 2011;21(8):880–886. 21306475 10.1111/j.1460-9592.2011.03532.x

[13] Karkouti K, Wijeysundera DN, Yau TM, et al. Acute kidney injury After cardiac surgery. Circulation. 2009;119(4):495–502. 19153273 10.1161/CIRCULATIONAHA.108.786913

[14] de Mendonça-Filho H, Pereira K, Fontes M, et al. Circulating inflammatory mediators and organ dysfunction after cardiovascular surgery with cardiopulmonary bypass: a prospective observational study. Crit Care. 2006;10(2):R46. 16542504 10.1186/cc4857 PMC1550915

[15] Kozik DJ, Tweddell JS. Characterizing the inflammatory response to cardiopulmonary bypass in children. Ann Thorac Surg. 2006;81(6):S2347–S2354. 16731102 10.1016/j.athoracsur.2006.02.073

[16] Yunus M, Karim HR, Saikia M, et al. Incidence and progression of cardiac surgery-associated acute kidney injury and its relationship with bypass and cross clamp time. Ann Card Anaesth. 2017;20(1):22–27. 28074790 10.4103/0971-9784.197823 PMC5290689

[17] Serraino GF, Provenzano M, Jiritano F, et al. Risk factors for acute kidney injury and mortality in high risk patients undergoing cardiac surgery. PLOS One. 2021;16(5):e0252209. 34019579 10.1371/journal.pone.0252209 PMC8139497

[18] Zappitelli M, Bernier PL, Saczkowski RS, et al. A small post-operative rise in serum creatinine predicts acute kidney injury in children undergoing cardiac surgery. Kidney Int. 2009;76(8):885–892. 19641482 10.1038/ki.2009.270

[19] Allegaert K, Smits A, van Donge T, et al. Renal precision medicine in neonates and acute kidney injury: how to convert a cloud of creatinine observations to support clinical decisions. Front Pediatr. 2020;8:366. 32850523 10.3389/fped.2020.00366 PMC7399072

[20] Li S, Krawczeski CD, Zappitelli M, et al. Incidence, risk factors, and outcomes of acute kidney injury after pediatric cardiac surgery: a prospective multicenter study*. Crit Care Med. 2011;39(6):1493–1499. 21336114 10.1097/CCM.0b013e31821201d3 PMC3286600

[21] Özker E, Saritaş B, Vuran C, et al. Early initiation of peritoneal dialysis after arterial switch operations in newborn patients. Ren Fail. 2013;35(2):204–209. 23176594 10.3109/0886022X.2012.745773

[22] Herbst C, Dworschak J, Schlager G, et al. Prophylactic peritoneal catheter placement in congenital cardiac surgery. World J Pediatr Congenit Heart Surg. 2022;13(3):376–378. 35446222 10.1177/21501351221084668

[23] Kher V, Sharma R, Bhan A, et al. Vasoactive inotrope score as a tool for clinical care in children post cardiac surgery. Indian J Crit Care Med. 2014;18(10):653–658. 25316975 10.4103/0972-5229.142174 PMC4195195

[24] Davidson J, Tong S, Hancock H, et al. Prospective validation of the vasoactive-inotropic score and correlation to short-term outcomes in neonates and infants after cardiothoracic surgery. Intensive Care Med. 2012;38(7):1184–1190. 22527067 10.1007/s00134-012-2544-x PMC4984395

